# Effect of forest-based biochar on maturity indices and bio-availability of heavy metals during the composting process of organic fraction of municipal solid waste (OFMSW)

**DOI:** 10.1038/s41598-023-42835-2

**Published:** 2023-09-25

**Authors:** Omid Hassanzadeh Moghimi, Gholamreza Nabi Bidhendi, Ali Daryabeigi Zand, Maryam Rabiee Abyaneh, Amir Nabi Bidhendi

**Affiliations:** 1 Department of Environmental Engineering, Kish International Campus, University of Tehran, Kish, Iran; 2 Faculty of Environment, University of Tehran, Tehran, Iran; 3 Department of Environmental Engineering, Aras International Campus, University of Tehran, Jolfa, Iran

**Keywords:** Environmental sciences, Environmental chemistry

## Abstract

The main objective of this study was to investigate the effect of biochar on the composting process of the organic fraction of municipal solid waste (OFMSW) under real conditions. Different doses of biochar (1%, 3%, and 5%) were mixed with compost piles to evaluate the variation of temperature, moisture content (MC), organic matter (OM), carbon (C), nitrogen (N), C/N ratio, and heavy metal (HM) contents in comparison with the control treatment (with 0% biochar addition). The results of this study showed that the compost piles combined with different doses of biochar had higher MC. The use of biochar as an additive, even at low doses (1%), was able to increase the compost quality through the reduction of N losses during the composting process. The highest reduction of OM during the composting process was observed in the control pile (without biochar addition) by 48.06%, whereas biochar affected the biodegradability of OM and prevented the reduction of nutrients during the composting process under real conditions. The contents of HMs (Pb, Zn, Ni, Cd, and Cu) showed a significant reduction in all of the compost piles combined with biochar in comparison with the control treatment. Considering that in terms of all compost quality indicators, the piles combined with biochar can regarded as high standard product, the composts obtained from combining the OFMSW with different biochar doses have desirable features to be used as an amendment agent to improve agricultural soil quality.

## Introduction

With the continuous growth of population and industry, global consumption has significantly increased, which has generated huge amounts of solid waste^1^. The resulting health and environmental problems especially in urban areas can ultimately give rise to great crises in human societies if not managed appropriately^2^. Many methods have been developed to dispose of the organic fraction of municipal solid waste (OFMSW). Composting is one of the most popular methods of recycling the OFMSW^3^. Compost production from OFMSW is conducted to reduce the volume and weight of disposable materials, decrease the smell and leachate, recycle resources, and mitigate potential disposal costs^4,5^. In composting method, organic waste is converted into a good fertilizer in agriculture, which has many advantages for soil amendment and applications as a nutrient supplement and conditioner for soil^6^. However, the use of compost in agriculture is limited due to its slow effect and the reduction of nutrient supply to agricultural products^7^. To improve the content of plant-absorbable nutrients in compost, its combination with nutrients and inoculation with microorganisms have been proposed^8^.

Biochar is a material rich in carbon (C) and nutrients, which is produced through the pyrolysis process^9^. The prominent characterizing features of biochar are the high surface area, porosity, and many other unique functions^10,11^. All these features make biochar an effective potential material in the optimization of the composting process and improvement of the final compost material^12^. During the composting process, the addition of biochar is effective in reaching a quicker compost maturity^13^, changing compost pH (usually with a decreasing effect)^14^, reducing the loss of nutrients such as calcium (Ca), magnesium (Mg), and nitrogen (N)^15^, increasing nitrification, forming stable humic materials^16^, reducing heavy metal (HM) mobility (lowering of their bioavailability)^17^, and decreasing greenhouse gas (GHG) emissions^18^.

Duan et al. (2021) addressed the effect of biochar on pollution control in the compost production process from sheep manure. It was reported that adding 7.5% biochar to the compost piles decreased the gaseous pollutants of ammonia (NH_3_), nitrous oxide (N_2_O), and methane (CH_4_) by 10.29 g, 0.47 g, and 30.29 g, respectively^19^. Wang et al. (2021) investigated the variation of N content during the composting process from distilled grain waste. The results demonstrated that biochar led to significantly lowered N loss during the composting process. The total N loss in the control treatment was 40.1%, which decreased to 25.69% by the addition of 10% biochar to the compostable materials^20^. Cui et al. (2020) addressed the mobility level and risk of HMs in the compost produced from swine manure and maize straw and stated that mixing 10% biochar with the piles reduced the concentrations of zinc (Zn), copper (Cu), cadmium (Cd), and lead (Pb) by 4.10%, 44.12%, 18.75%, and 30.06%, respectively compared to the control^21^.

The effect of mixing biochar with compostable materials on the quality of compost produced from municipal solid waste was investigated in various researches^1,18,22,23^. Unlike other researches that were mainly conducted on a laboratory scale, the present research was conducted in order to investigate the effect of adding biochar on the composting process of solid organic waste materials on an industrial scale (using mechanical processing, fermentation in a closed space and mechanized aeration). On the other hand, wastes of green spaces are an important part of the composition of urban solid wastes, which, in addition to the problems caused by the formation of greenhouse gases, impose a lot of costs on city administrations to organize this part of wastes. Therefore, the use of forest wastes for biochar production in this research can be considered as a solution for managing this group of wastes (along with the wastes from urban green spaces) and can be considered as one of the effective results of this research. Due to conducting this research in the industrial phase, the current research can be the beginning of new paths for investigating and feasibility of adding biochar as a supplement in the composting process of urban organic solid waste materials on an industrial scale. Among the other results of this research, we can mention the creation of productivity and economic efficiency in the industrial production of compost, the reduction of waste management costs, and the control and reduction of environmental effects caused by the accumulation of urban waste and forest waste. The aim of the research was to assess whether and how the biochar application at different doses affected the course of the composting process (thermophilic phase) and the quality of the compost. Furthermore, the assessment planned to observe how the physicochemical properties of waste (temperature, moisture content (MC), organic matter (OM), N, and C/N ratio) would change. Additionally, the final product was analyzed for the content of some HMs (Pb, Zn, Ni, Cd, Cr, and Cu) essential in terms of the compost assessment.

## Materials and methods

### Composting feedstock collection

In the present study, the composting process from OFMSW was carried out under real conditions in the Composting Facility of the Babol Municipality (CFBM), Mazandaran province, Iran. With features such as mechanized processing technology, fermentation under a roofed enclosure, and screw mixer, this facility converts OFMSW of different districts of Babol and the surrounding villages to compost with the goal of lowering environmental pollution and improving waste management practices. Currently, about 250 tons of wastes are transported to the CFBM on a daily basis, which consists of food waste (65.3%), paper and cardboard (8.7%), polyethylene terephthalate (PET) (1.1%), rubber (0.5%), plastic (7.3%), textile (1.1%), glass (1.2%), ferrous metals (1.8%), non-ferrous metals (2.3%), wood (1.2%), and others (9.5%)^24^. As can be seen, more than 65% of waste produced in Babol is composed of degradable waste, which has the ability to be converted into compost.

### Biochar preparation and determination of physical and chemical characteristics

The biochar used in this study was purchased from Beshel Biochar Facility which is located in Mazandaran, Iran. The biochar was produced from a mixture of forestry residues (including the wood waste of maple, beech, and alder trees) using the pyrolysis process at 700 °C during 8 h. Table 1 shows the physical and chemical properties of the biochar. C content is the most important constituent of various biochars. In this regard, considering a high percentage of C in the biochar sample used in this study (76.10%), it was expected that this material would have a good performance during the research process. The low amount of N in the biochar (0.71%) occurs due to N losses in the form of NH_3_ and/or nitrogen oxides (NOx) during the carbonization process. In addition, the specific surface area of the biochar sample used in this research was 340 m^2^/g, indicating its porous structure.

**Table 1:** Physicochemical properties of the biochar used in the compost production process.

Properties	Unit	Value
Pyrolysis temperature	°C	700
Retention time	hour	8
Particle size	mm	1.18–2.36
pH-	–	8–8.53
MC	%	6.00
C	%	76.14
N	%	0.71
BET surface area	m^2^/g	340

*MC* moisture content, *C* carbon (in dry mass), *N* nitrogen (in dry mass).

### Composting process

The composting process lasted for 9 weeks (63 days), which started from Saturday, June 12, 2021 and ended on Friday, August 13, 2021. Weather conditions in the research area were stable during the composting process, and since the reception, residence, and processing halls of the CFBM were roofed, the compostable materials were protected from rain, moisture, or other environmental and weather phenomena. Present research aims to evaluate the effect of small selected doses of biochar addition on the composting process of the OFMSW. So, three different doses of biochar were selected and added to the compost piles, 1%, 3%, and 5% (windrows with a width and height of 1 m and a length of 3 m) and 0% without the addition of biochar as a control sample (% are expressed as wet weight). Mixing biochar and compostable materials in the covered halls of the fermentation unit homogeneously using a loader and a box turner (a type of mechanical screw turner with a structure similar to a reclaimer installed on a stacker and capable of moving in length and width has the fermentation hall) is done.

The compostable materials that have been used to conduct this research are the perishable materials in the mixture of urban mixed waste entering the compost production plant, which are mechanically processed in the processing unit (using a trommel screen with an opening diameter of 2.5 m, a length of 12 m and the diameter of the outlet holes is 60 mm) separated and transferred to the fermentation unit. These materials are arranged in windrows with width and height of 1 m and length of 3 m. Taking into account the density of the subsoil material (470 kg/m^3^), the weight of each windrow is about 550 kg and its volume is 1.17 m^3^.

In the thermophilic composting phase, i.e., the first three weeks, the aeration of the compost piles were performed at an air current rate of 0.2 m^3^/min in a periodic manner. Temperature feedback was scanned using an automatic control system. Continuous aeration was performed in the first 12 h, and then, the system was turned on at 65 °C and turned off at 60 °C. This temperature range was controlled using a programmable logic controller. The compost piles temperature was measured using a ThermoHygrometer device equipped with a probe, which made it possible to measure the temperature in piles at a depth of 1 m. The temperature was measured twice a day. When the composting process completed at the end of week nine, the compost piles were transported to the final processing hall and sifted and prepared using a trommel screen (with 20-mm mesh), vibrating screen, and glass separator.

### Sampling and laboratory analysis

During the bio-oxidative process, the thermophilic phase is considered to be the most effective stage for the biodegradation of organic matter in municipal solid waste. Gradual temperature increases to the thermophilic stage (40–60 °C) over the three-week composting process, indicating microbial breakdown of organic solid waste. Therefore, the sampling of the OFMSW was conducted at five points in time, namely 0, 7, 14, 21, and 63 days from the start of the composting process. Samples were collected in three replications. Before and after each sampling, a loader fully blended the compost piles. At each sampling point, samples were taken from the center of the pile. The collected samples were then poured into a clean plastic bucket. Once the sampling ended, materials in each bucket were completely mixed to avoid the formation of any layer based on the particle size. In total, 60 samples consisting of 15 samples from the control treatment (without biochar addition) and 45 samples from the composting piles mixed with different doses of biochar were collected and immediately transferred to the laboratory. After preparing the samples, their pH, MC, OM, C, N, and HMs concentrations were measured.

The pH of the samples was measured via the procedure below: sample extraction using a horizontal shaker with water at solid-water ratio of 1:10 (dw/v) for 3 h at 200 rpm at ambient temperature (25 °C), centrifugation at 3000 × *g* for 20 min, and filtration with 0.45 µm syringe filters. Following the shaking step, a smart CHEM-LAB Laboratory Analyzer was used to determine pH in an electrometrical manner in slurry. To determine the MC, the samples were oven-dried at 105 ± 2 °C until reaching a constant weight. The loss on ignition of dried and ground mass was determined after heating in the muffle furnace at 550 ± 15 °C for 4 h and used to obtain the OM. The C content was determined using an ELTRA CHS-580A analyzer in a furnace preheated to 1350 °C, where the sample was burnt and flue gas was directed to the measuring cuvettes. ELTRA CHS-580A analyzers is equipped with an automatic sample loader and is the ideal analyzer for the determination of C, hydrogen (H) and sulfur (S) in organic samples. The CHS-580A is based on the technology of the CHS-580 analyzer and features a vertical resistance furnace with ceramic tube. To determine the N content, an ELTRA N 580 analyzer was employed in an oven preheated to 950 °C. Equations (1), (2) developed by Paredes et al. (1996) were used to calculate the OM and N loss values according to initial (X_1_) and final (X_2_) ash contents^25^.

1 $$OM\, loss\, \left(\%\right)=100-100\frac{{X}_{1}\left(100-{X}_{2}\right)}{{X}_{2}\left(100-{X}_{1}\right)},$$

2 $$N\, loss\, \left(\%\right)=100-100\frac{{X}_{1}{N}_{2}}{{X}_{2}{N}_{1}},$$

where N_1_ and N_2_ are the initial and final N concentrations, respectively.

The ICPOES model 5100 SVDV inductively coupled plasma mass spectrometry system was employed to obtain HMs concentrations in dry mass (d.m.). The sample preparation procedure consisted of an Aqua Regia (AR) digestion on a hot plate. 1.0 g sample in 12 mL AR was refluxed at 60 °C for 0.5 h and then at 110 °C for 2 h. The mass in the known volume was used to determine wet bulk density.

### Statistical analysis

Statistical analysis of the obtained results was made using the IBM SPSS-v.21 software package. One-way analysis of variance (ANOVA) and least significant difference (LSD) at p < 0.05 were performed in order to check the significance of the selected physicochemical properties and HMs contents in samples collected at different times from piles with different biochar contents.

## Results and discussion

### Physicochemical properties of materials used for the composting experiments

The results obtained from the analysis of the physicochemical characteristics and HMs concentrations in initial materials (OFMSW) used for the composting process are provided in Table 2. Based on which, the pH value of OFMSW is 7.88 ± 0.1, which puts it in the alkali range. Awasthi et al. conducted similar research in the composting facility of Jabalpur, India, and reported that the pH value of the raw materials was in a similar range and equal to 7.27^26^. Furthermore, the initial pH of the piles in a composting facility in Beijing, China, was reported by Zhang et al. as 7^27^.

**Table 2:** Physicochemical properties of raw materials used for the compost production.

Properties	Unit	Value
MC	%	59.20 ± 2.33
OM	% d.m	50.48 ± 3.10
Bulk density	kg/m^3^	519.14 ± 32.55
C	% d.m	31.02 ± 6.54
N	% d.m	2.18 ± 0.27
C/N	–	14.23 ± 1.85
pH	–	7.88 ± 0.14
Cd	mg/kg d.m	1.02 ± 0.38
Cr	mg/kg d.m	35.50 ± 1.72
Cu	mg/kg d.m	299.94 ± 18.19
Ni	mg/kg d.m	46.15 ± 39.06
Pb	mg/kg d.m	101.78 ± 7.33
Zn	mg/kg d.m	1497.96 ± 35.44

Mean ± standard error of mean (n = 3).

*MC* moisture content, *OM* organic matter (in dry mass), *C* carbon (in dry mass), *N* nitrogen (in dry mass).

Table 2 indicates that the MC of OFMSW is 59.20 ± 2.3%, which is in compliance with the moisture range obtained by Graça et al. for the initial materials in a compost production line of a facility in Ireland (58.7%)^1^. On the other hand, Kepa Izaguirre et al. reported that the MC of OFMSW of Gipuzkoa, Spain, was 41%^28^. This difference among the reported MC is mainly a result of different compositions of organic waste materials and climatic and atmospheric conditions of the regions of interest^29,30^. Analyzing the characteristics of initial materials in this research indicates lower OM (50.48 ± 3.1% d.m.) in comparison with the values reported in other countries, especially developed countries^31–33^. Difference among the reported values is generally related to the composition of compostable materials, which mainly arises from the implementation of separate collection systems and different efficiencies of material separation and processing systems in composting facilities^34,35^.

The measured HMs concentrations in OFMSW were reported in Table 2 show that the concentrations of some HMs such as Zn, Cu, and Pb are 1497.96 ± 35.4 mg/kg d.m., 299.94 ± 18 mg/kg d.m., and 101.78 ± 7.3 mg/kg d.m., which are relatively high. Asquer et al. (2017) reported that the concentrations of Cu and Zn were respectively 17.76 mg/kg d.m. and 56.64 mg/kg d.m.^36^, which are significantly smaller than the mean concentrations of these metals in OFMSW of the present research. These different reported values are mainly attributed to differences between the collection and transportation methods of municipal solid waste (MSW)^37^. Given the lack of source separation of MSW in Babol, which leads to the delivery of the waste in the mixed state to the CFBM, substances including batteries, dyes, electronic devices, plastics, newspapers, domestic cleaning products, cosmetics, packaging, and medications are present in the initial materials and thus increase the concentration of HMs in them.

### Biochar effect on composting performance

#### Temperature

**Figure 1:**
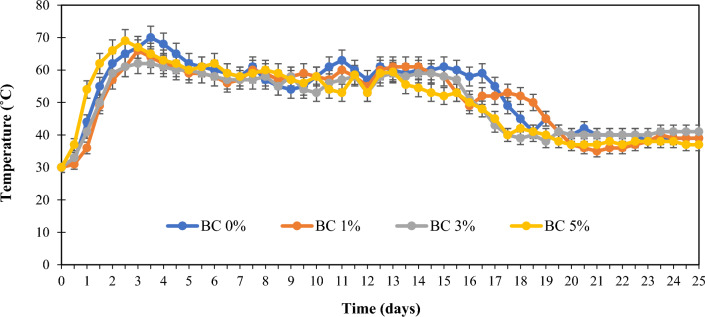
Evaluation of temperature in different treatments during composting of OFMSW with biochar addition. Control pile BC 0%: OFMSW without biochar addition; BC 1%: OFMSW + 1% biochar; BC 3%: OFMSW + 3% biochar; BC 5%: OFMSW + 5% biochar.

Temperature variation during the composting process in the control pile and the composting piles containing biochar is shown in Fig. 1. In the CFBM, thermophilic temperature (above 45 °C) was observed in the piles between days 2 and 16 of the composting process. With the start of the composting process, the temperature of the materials increased at a relatively high rate to the extent that 30 h after the start of the composting process, the temperature of the control pile reached around 60 °C. The maximum temperature in the control pile was 70 ± 2 °C, which was observed on days 3 and 4 from the start of the composting process. Comparing the results with those of similar studies conducted on the composting process of OFMSW in real conditions shows that the thermophilic phase in the present research ended comparatively faster^38,39^. Between days 20 and 22 of the composting process, the temperature of materials in the piles declined and reached 40 ± 3 °C and remained at this level until the end of the composting process on August 11, 2021. No significant change in temperature occurred in all the tested piles in the composting process, and the addition of biochar to the materials had no notable effect on the temperature variation of these materials; the only observed change occurred in the pile containing 5% biochar, in which a temperature of above 60 °C was obtained 20 h after preparing the piles and starting the composting process (which on average occurred 8 h sooner than other piles under consideration). Note that in similar research conducted by Nguyen et al., the ineffectiveness of biochar on the temperature during the composting process is confirmed^40^. However, the results of research by Xiao et al., Ravindran et al., and Malinowski showed that the addition of biochar to OFMSW for compost production makes the thermophilic phase slightly longer^41–43^.

At the beginning of the composting process, mesophilic bacteria with a temperature activity range of 45–20 degrees Celsius are dominant. Then, with the start of the thermophilic phase, bacteria with an activity range of 45–75 degrees Celsius prevail. In the cooling stage, mesophilic bacteria start to work again and in the last stage (compost maturation stage), actinomycetes, molds and some fungi appear^38^. Specifically, an excessive and continuous increase in temperature (typically more than 65 degrees Celsius), leads to a decrease in the population of beneficial microorganisms and, as a result, a decrease in the speed of the composting process^3^ and a decrease in temperature leads to a decrease in the activity of effective microorganisms and the reduction of production efficiency will occur^18^. It is very necessary and vital that during the active stage of industrial composting, the temperature is kept within the range of activity of thermophilic bacteria so that pathogens and weed seeds are destroyed. The most appropriate way for this purpose is to use mechanical aeration methods^8^.

#### Moisture content

Characteristics of the MC and the other parameters measured during the process are shown in Table 3. The MC at the beginning of the process in the control compost pile was 59.20 ± 2.3%, which declined with time as the aeration operation continued until it reached 32.21 ± 2.8% on the last sampling day (day 63). Similar variation was observed in the compost piles containing biochar. The evaporation of moisture during the process lowered the MC of the pile with time. On the other hand, the presence of biochar in different compost piles had no significant effect on the moisture retention; in addition, MC difference between the piles containing biochar and the control pile was not statistically significant. The only exception was the significant difference in the measured MC between the control pile and the pile containing 5% biochar at the end of the composting process. During the process, in addition to the production of carbon dioxide (CO_2_) in the aeration process, water is also produced^44^. It seems that in the pile containing 5% biochar, increase in the MC due to the formation of water compensated for the moisture loss due to evaporation, and thus, a lower reduction trend is seen in this pile compared with other piles. Ravindran et al. and Malinowski and Famielec reported that the high-water retention capacity of biochar due to its porous structure leads to the absorption of moisture from composting piles^42,45^.

**Table 3:** Evaluation of MC, OM, C, N, and C/N ratio in different piles during composting of OFMSW with biochar addition.

Composting process (days)	MC (%)	OM (% d.m.)	C (% d.m.)	N (% d.m.)	C/N
BC 0%: OFMSW without biochar addition (control pile)					
0	59.20 ± 2.37	50.48 ± 2.93	31.02 ± 0.75	2.18 ± 0.23	14.23 ± 1.71
7	51.93 ± 1.91	49.98 ± 5.08	27.45 ± 2.39	2.18 ± 0.14	12.59 ± 1.36
14	49.93 ± 1.74	45.03 ± 3.54	25.90 ± 1.90	2.18 ± 0.10	11.88 ± 1.05
21	48.95 ± 3.99	39.85 ± 1.17	20.72 ± 1.71	1.91 ± 0.21	10.85 ± 1.59
63	32.21 ± 2.85	26.22 ± 3.14	17.56 ± 0.62	1.65 ± 0.24	10.64 ± 1.24
BC 1%: OFMSW with 1% biochar addition					
0	58.50 ± 2.56	51.14 ± 3.36	32.83 ± 0.95*	2.49 ± 0.23	13.18 ± 2.16
7	58.50 ± 2.80	48.70 ± 2.16	27.35 ± 1.72	2.20 ± 0.11	12.43 ± 0.93
14	53.67 ± 3.92	47.75 ± 1.91	26.30 ± 0.97	2.05 ± 0.12	12.83 ± 1.45*
21	50.16 ± 3.44	44.21 ± 3.80	26.17 ± 0.94*	2.05 ± 0.20	12.77 ± 2.78*
63	38.58 ± 3.87	30.70 ± 3.65*	24.46 ± 0.83*	1.92 ± 0.23*	12.74 ± 1.19*
BC 3%: OFMSW with 3% biochar addition					
0	57.88 ± 2.91	53.25 ± 4.90	32.90 ± 2.15*	2.19 ± 0.20	15.02 ± 2.30*
7	55.65 ± 3.65	45.13 ± 4.87	29.37 ± 1.18*	2.19 ± 0.21	13.41 ± 1.44*
14	52.01 ± 1.82	42.58 ± 2.12	28.24 ± 2.09*	1.92 ± 0.22	14.71 ± 2.36*
21	50.99 ± 3.11	42.15 ± 5.15	24.34 ± 0.86*	1.92 ± 0.13	12.68 ± 1.17*
63	39.22 ± 1.45	32.43 ± 4.24*	23.41 ± 1.02*	1.92 ± 0.20*	12.19 ± 2.53*
BC 5%: OFMSW with 5% biochar addition					
0	57.63 ± 2.13	54.31 ± 4.61	34.38 ± 3.35*	2.51 ± 0.25	13.7 ± 2.09
7	55.49 ± 2.76	50.76 ± 4.95	31.25 ± 1.24*	2.36 ± 0.22	13.24 ± 1.73*
14	52.37 ± 3.63	49.28 ± 4.08	29.21 ± 0.63*	2.36 ± 0.20	12.38 ± 0.85
21	49.88 ± 3.35	44.39 ± 2.40	26.31 ± 1.41*	2.20 ± 0.13	11.96 ± 1.64*
63	45.76 ± 4.22*	34.95 ± 2.22*	25.06 ± 1.77*	2.06 ± 0.12*	12.17 ± 0.81*

Mean ± standard error of mean (n = 3).

*MC* moisture content, *OM* organic matter (in dry mass), *C* carbon (in dry mass), *N* nitrogen (in dry mass).

*Significant at p < 0.05.

#### Organic matter

According to Table 3, the highest content of OM is observed in the pile containing 5% biochar (34.95 ± 2.2% d.m.). In contrast, the highest reducing trend of OM during the composting process was observed in the control pile (48.06%), such that at the end of the process, the content of OM in it was measured as 26.22 ± 3.1% d.m. In the piles containing 1%, 3%, and 5% biochar, OM declined by 39.97%, 39.1%, and 35.65%, respectively, during the process. Difference in the final loss of the OM between the control pile and those containing biochar is statistically significant. This suggests that biochar can affect the decomposition rate of OM and prevent the reduction of nutrients during the composting process in real conditions which was also confirmed by other studies. Nguyen et al. reported that the decrease in the OM losses in the piles with higher rates of biochar can somewhat attributed to the ratio of biochar added to the piles and the recalcitrance of biochar towards microbial degradation^40^. Similar results were obtained by An et al. (2012) and Waqas et al. (2018) but the situation may differ if high proportions of the biochar are added^30,46^. Similar results were obtained by Chaher et al. (2020) who added biochar at a high dose (20% in volume), reported 47% OM loss during the composting process^47^.

As mentioned in the results of the research, biochar did not have a clear effect on temperature changes during the composting process; In such a way that with the beginning of the composting process, the temperature increased and after the third week, it decreased and remained almost the same until the end of the composting process. Despite the fact that the maximum increase in temperature was before the 20th day (during the thermophilic process), the greatest decrease in the humidity parameter occurred after the 20th day. The reason for this contradiction is that the process of reducing humidity is not only related to temperature changes, but the mechanized process of aeration from the floor of the fermentation hall during the process has also been effective in the process of changes in humidity. Mechanized aeration from the bottom of the windrow, in the first 12 h of the composting process, continuously and periodically during the thermophilic phase at a rate of 0.2 cubic meters per minute, with the automatic turning on of the aeration fans at a temperature of 65 degrees Celsius and their automatic shutdown at The temperature of 60 degrees Celsius has been done and as a complementary factor along with the temperature factor, it has recorded the highest trend of reducing the moisture content of the compost pile after the 20th day. The highest decrease in organic matter after the 20th day was recorded due to the activity of mesophilic bacteria in conditions of low humidity, which affects the process of microbial decomposition of compostable materials. Also, with the completion of the thermophilic period and the completion of the biological decomposition cycle, biochar can play a role in the reduction process of organic materials by affecting the rate of decomposition and degradability of organic materials in addition to reducing the temperature factor.

#### Nitrogen

According to Table 3, the highest N loss was 24.32%, which was measured in the control pile. In the piles containing 1%, 3%, and 5% biochar, the N loss values are 22.9%, 12.33%, and 17.93%, respectively. Difference in the final N loss between the control pile and the piles containing biochar is statistically significant but lower then was demonstrated by Oviedo-Ocaña et al. (2015) (a 27–34% higher retention of N compounds in the compost piles containing biochar compared to the compost without biochar)^48^. Furthermore, Godlewska et al. (2017) reported that amending biochar with the composting piles increased the content of N in the final product since the reduction trend of N declines in the pile containing biochar during the composting process due to the adsorption of volatile NH_4_^+^ and/or NH_3_ at the biochar surface area^44^.

#### Carbon

The variation pattern of the C content of the composting piles (Table 3) demonstrates a considerable reduction of C (by 43.4%) in the control pile, similarly as in the case of OM content. In the composting piles amended with 1%, 3%, and 5% biochar, the C declined by 25.5%, 28.85%, and 27.11%, respectively, all of which were lower compared with that of the control pile. The results of a similar research by Waqas et al. (2018) showed that the C content of the compost pile without biochar was about 40% during the 90-day composting period; however, the C content of the compost piles containing 10% and 15% biochar declined by 20–40%^30^. In another study, Tessfaw et al. (2020) reported that after the end of the 88-day period of composting process from OFMSW, the C content in control pile (without biochar addition) was measured as 16.76% d.m., while in the piles containing biochar, loss in the C content was lower. At the end of the composting process, the C content of the piles containing 15% and 25% biochar were measured as 19.38% d.m. and 21.45% d.m., respectively^3^, which is similar to the trend seen in the present study.

#### The C/N ratio

As can be seen in Table 3, C/N ratio in the control pile was 14.23 ± 1.7%, which declined by 25.23% after 60 days and reached 10.64 ± 1.2%. During the composting process, C/N ratio decreased almost uniformly as the consumption of C by microorganisms as an energy source increased for both building cellular texture and producing various N compounds during the composting process^20,49^. Awasthi et al. (2014) examined the compost production process from MSW and reported that at the end of the 35-day period of the process, C/N ratio declined by 24.26%^50^. Moreover, in a study by Fourti (2013) on the maturation of the compost produced from MSW of a city in northern Tunisia, the use of windrow composting for 120 days lowered the C/N value from 32 to 14.6%, indicating a 54.37% decline^51^.

#### Heavy metals

**Figure 2:**
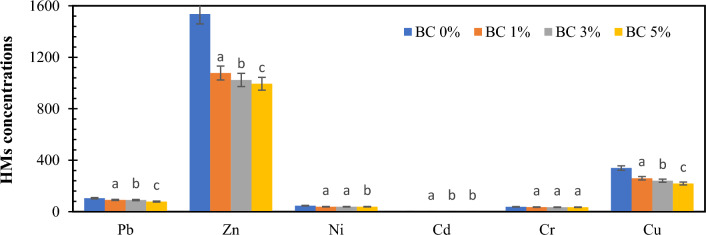
HMs concentrations in mature composts from different piles of OFMSW with biochar addition. BC 0%: OFMSW without biochar addition; BC 1%: OFMSW + 1% biochar; BC 3%: OFMSW + 3% biochar; BC 5%: OFMSW + 5% biochar. Different letters above the bars indicate statistically significant difference at p < 0.05.

Figure 2 shows the HMs concentrations of the composts at the end of the 9-week research cycle. The control pile and the pile containing 5% biochar have respectively the highest and the lowest concentrations of HMs among the compost treatments. The concentrations of Pb, Zn, Ni, Cd, and Cu were significantly decreased in all the compost piles with biochar (piles 2, 3, and 4) compared with those of the control pile (without biochar addition). On the other hand, no significant difference observed for Cr concentrations in different compost piles. Among different HMs, Cd showed the highest reduction compared with the control pile with 71.63% reduction in the pile containing 5% biochar. Moreover, the concentration of this metal in the pile with 1% and 3% biochar decreased by 55.82% and 70.24%, respectively, compared with that in the control pile. It is also seen that the concentration variations of Cd in the piles with 3% and 5% biochar are close to each other. Similar observation was also seen for Cu and Zn; in this regard, increasing the biochar content from 3 to 5% in the composting piles did not lead to further reduction in the concentrations of these metals at the end of the process. The concentration variation of Ni in the piles containing biochar was entirely different from those of other HMs. In the control pile, the concentration of this metal was 46.19 ± 2.3 mg/kg d.m., while in the piles with 1%, 3%, and 5% biochar, it decreased to 38.49 ± 2 mg/kg d.m., 38.49 ± 1.7 mg/kg d.m., and 37.47 ± 2.3 mg/kg d.m., respectively. The reduction of Ni concentration in the piles with 1% and 3% biochar was 16.68%, while this reduction in the pile with 5% biochar was 16.72%. As can be seen, no positive effect was seen on the reduction of Ni concentration as the content of biochar amended with the composting piles increased. An increase in the content of trace elements in biologically processed materials without the addition of biochar was confirmed by other authors^19,21^. In the case of biological processing, the reduced OM content in the processed biomass led to the increased content of HMs^10^.

Comparison of the physicochemical properties of the composts produced after the 9-week research cycle with the Iranian compost quality standards^52^ demonstrates that most of the physicochemical properties of compost in control pile (i.e., MC, OM, C, C/N) are exceeded to compost standards. This is while the composts produced from the piles which amended with biochar have acceptable values for all compost quality indicators. In terms of important parameters of composting process i.e., pH, MC, OM, C, N, C/N ratio, and HMs content, all the three piles containing biochar (1%, 3, and 5%) are in the standard range, and the compost combined with 5% biochar is in more desirable conditions in this regard. Therefore, the composts produced from combining OFMSW with different doses of biochar can be used as a useful amendment for soil fertilizer.

Many studies investigating composting processes with various initial materials have reported reduced concentrations of HMs in the final product of compost samples containing biochar compared to compost samples without biochar. Duan et al. (2021) analyzed the concentrations of HMs during the composting process from sheep manure and stated that Zn concentration in the control treatment (without biochar addition) was 211.9 mg/kg d.m. while its concentrations in compost treatments with 2.5%, 5%, 7.5%, 10%, and 12.5% biochar were 186.47, 175.71, 171.04, 164.87, and 161.458 mg/kg d.m., respectively^19^. According to the findings of another study, amending 1.5% and 5% biochar with OFMSW as the composting material led to 49.5% and 55.72% reduction in the Zn and Cd, respectively^38^. Furthermore, Wang et al. (2022) reported that adding biochar in the composting process from swine manure reduced the concentration of Zn and Cu by 9.9% and 24.8%, respectively^53^.

The results of previous studies have determined that biochar shows a significant correlation with heavy metals^19^. Biochar is used to purify water environments and immobilize heavy metals in soil and sediment^10^. Therefore, it can be concluded that the addition of biochar to compostable materials during the production process also leads to the stabilization of heavy metals in the final product. Due to its porous structure, high specific surface and many functional groups, biochar can absorb heavy metals in compost and reduce their mobility through the following mechanisms^21^:

- Physical absorption by biochar.
- Formation of complex bonds on the surface of biochar (between biochar and heavy metals).
- Ion exchange between heavy metals and ions in the biochar structure.
- Electrostatic interactions at the biochar level.
- An increase in pH (which leads to an increase in the precipitation of heavy metals and a decrease in their mobility).
- Electrostatic reactions on the surface of biochar.

### Cost of biochar produced from forestry wastes

The cost of biochar production is a key component in the marketing and application of biochars and significantly impacts upon the overall cost (operating and capital) of any composting technologies. The overall cost for the preparation of 1 kg is estimated to be 0.841 US$/kg (841 US$/ton) which were estimated by the manufacturer, Beshel Biochar Facility (http://beshelactivatedcarbon.ir). According to current information, the cost of commercially available carbonous adsorbents in the world market varies between ~ 800 and 5000 US $/ton (depending on the quality/type of adsorbent)^54,55^.

Based on the estimate made for managing the industrial production line of compost from urban solid waste materials (taking into account personnel costs, maintenance and repair of equipment and machines and other related costs), the cost of producing one kilogram of compost is 1 cent. And its selling price is 5 cents per kilogram without forming and enriching process and in bulk. If the compost produced at the end of the production process is shaped in the form of powder, granules or pellets and is enriched by injecting the required nutrients and has a suitable packaging, its selling price will be higher. According to the proof of the positive effect of using biochar on the quantity and quality of compost produced in this research and taking into account the cost of production and selling price of compost and the cost of producing biochar, the sale of compost modified with biochar can lead to economic profitability.

## Conclusions

The main innovation of the current research was to show the effect of adding biochar on the composting process of solid organic waste materials on an industrial scale (using mechanical processing, fermentation in a closed space and mechanized aeration). Improving the properties of compost production, increasing the speed of the industrial production process (through a gradual decrease in temperature at the end of the process), reducing pH, increasing humidity, stabilizing organic matter, reducing the rate of nitrogen reduction, improving the C/N ratio and reducing the concentration of heavy metals due to the addition Biochar to compostable materials is one of the specific results of this research.

The use of forest wastes for biochar production in this research can be considered as a solution for managing this group of wastes (along with the wastes from urban green spaces) and can be considered as one of the effective results of this research. Due to conducting this research in the industrial phase, the present research can initiate new opportunities for investigating and feasibility of adding biochar as an additive in the composting process of urban organic solid waste materials under industrial conditions and lead to the creation of productivity and economic efficiency.

## Data Availability

The datasets generated during and/or analyzed during the current study are available from the corresponding author on reasonable request.
